# The efficacy of three antimicrobial dressings in the management of diabetic foot ulcers: a systematic review and network meta-analysis

**DOI:** 10.3389/fendo.2026.1780580

**Published:** 2026-02-24

**Authors:** Xiaoyuan He, Yaoxing Li, Yunxiu Chen, Junhua Lu, Wei Zhang, Mei Ning, Zhenguo Han

**Affiliations:** 1 School of Nursing, Mudanjiang Medical University, Mudanjiang, Heilongjiang, China; 2 Department of General Practice, Hongqi Hospital Affiliated to Mudanjiang Medical University, Mudanjiang, Heilongjiang, China; 3 Department of Respiratory Diseases, The Second Affiliated Hospital of Mudanjiang Medical University, Mudanjiang, Heilongjiang, China; 4 General Surgery Department, Hongqi Hospital Affiliated to Mudanjiang Medical University, Mudanjiang, Heilongjiang, China; 5 General Surgery Department, Shanxi Bethune Hospital, Taiyuan, Shanxi, China

**Keywords:** antibiotic bone cement, diabetic foot ulcers, honey dressings, network meta-analysis, silver ion dressings

## Abstract

**Background:**

Antimicrobial dressings have been used to treat diabetic foot ulcers (DFU). However, no direct or indirect comparison among silver dressings, honey dressings or antibiotic-impregnated bone cement (ABC) for DFU has been performed to date.

**Objective:**

To systematically evaluate the efficacy of the three antimicrobial dressings in the management of DFU.

**Methods:**

A comprehensive search of PubMed, Embase, The Cochrane Library, and Web of Science was conducted to identify randomized controlled trials (RCTs) comparing silver dressings, honey dressings, and ABC in the treatment of DFU, from database inception to November 2025. Two investigators in-dependently performed study selection, data extraction, and assessment of risk of bias. Data analyses were conducted using Stata 18.0.

**Results:**

A total of 15 RCTs encompassing 1,292 patients were included. The network meta-analysis revealed that ABC demonstrated superiority over standard care in both healing rate and healing time (OR = 4.06, 95% CI [1.48, 11.1], P < 0.05; SMD = –2.25, 95% CI [–4.11, –0.40], P < 0.05). Silver dressings also outperformed standard care in terms of healing rate and wound area reduction (OR = 2.31, 95% CI [1.14, 4.69], P < 0.05; SMD = 1.43, 95% CI [0.39, 2.47], P < 0.05). Based on the surface under the cumulative ranking curve, ABC and silver dressings ranked as the most favorable therapeutic options.

**Conclusions:**

According to the network meta-analysis and SUCRA ranking results, an-tibiotic bone cement and silver dressings appear to be the most advantageous strategies for DFU management. Future studies should include direct comparisons among various antimicrobial dressings and incorporate cost-effectiveness analyses to guide optimal clinical decision-making.

**Systematic review registration:**

https://www.crd.york.ac.uk/PROSPERO/view/CRD42024578370, identifier CRD42024578370.

## 1 Introduction

Diabetes represents a prominent global health burden. It is estimated that 589 million adults were living with diabetes worldwide in 2024, among whom nearly half remained undiagnosed. Furthermore, approximately 1.1 billion adults globally exhibited impaired glucose tolerance or impaired fasting glycaemia, conditions that confer an elevated risk of developing type 2 diabetes mellitus (T2DM). Projections indicate that these prevalence figures will rise substantially over the next two decades (1). Diabetic foot ulcer (DFU) is among the most prevalent complications of diabetes (2), and its impaired healing is frequently associated with tissue ischemia, neuropathy, and infection (3), imposing a substantial economic burden on patients and their families(4). Consequently, effective wound management is of paramount importance for optimizing outcomes in individuals with DFU. At present, the treatment of DFU primarily consists of comprehensive strategies such as offloading, debridement, and infection control (5), among which infection control may involve both systemic and topical antimicrobial therapies. The use of antimicrobial dressings constitutes a major approach to topical infection management.

In clinical practice, the primary function of antimicrobial dressings is to reduce bacterial burden and to facilitate angiogenesis, thereby accelerating wound healing. Silver dressings, honey dressings and ABC have been increasingly applied in the management of diabetic foot ulcers. Silver dressings represent a novel class of functional wound dressings with broad-spectrum antimicrobial activity. They are capable of suppressing inflammatory responses and facilitating wound repair (6). Honey dressings enhance the healing process by promoting angiogenesis, granulation tissue formation, and re-epithelialization. Multiple studies have demonstrated significant clinical benefits of silver dressings (7, 8) and honey dressings (9) in patients with DFU. Antibiotic-impregnated bone cement (ABC) was initially introduced for managing infections following joint arthroplasty (10) and serves a dual purpose: preventing soft-tissue contracture and delivering high local concentrations of antibiotics to bone and soft tissues via controlled elution (11). In recent years, its application has been extended to patients with DFU, where it has shown promising antimicrobial properties and favorable wound-healing outcomes (12). Despite these advancements, the comparative efficacy, safety, and relative advantages of these three antimicrobial modalities in the treatment of DFU remain uncertain. Therefore, the present study employed a network meta-analysis to evaluate the therapeutic effectiveness of silver dressings, honey dressings, and ABC in DFU management, and to explore their prospects and limitations, with the aim of providing robust and evidence-based guidance for clinical practice.

## 2 Methods

This study followed the Preferred Reporting Items for Systematic Reviews and Meta-Analyses (PRISMA) 2020 guidelines for its conduct and reporting (**Supplementary Tables 1**, **2**.). This review was registered in the International Prospective Register of Systematic Reviews (PROSPERO) on 10 August 2024 (registration number: CRD42024578370). Institutional review board approval was not required for this study.

### 2.1 Search strategy

A comprehensive literature search was conducted in PubMed, Embase, The Cochrane Library, and Web of Science from database inception through November 2025. Additionally, the references of relevant published systematic reviews, meta-analyses, and included studies were manually screened to identify potentially missed studies. Searches combined both Medical Subject Headings (MeSH) and free-text terms. Detailed search strategies are presented in **Supplementary Table 3**.

### 2.2 Selection criteria

Title/abstract and full-text screening were conducted independently and in duplicate by investigators (X.H. and S.C.), with disagreements resolved by discussion or adjudication by a third researcher. We included (1): Study Design: randomized controlled trials (RCTs) written in English (2). Participants: (a)Adult patients with a confirmed diagnosis of type 1 or type 2 diabetes mellitus; gestational diabetes was excluded; (b)Patients meeting the 2023 International Working Group on the Diabetic Foot (IWGDF) criteria for DFU; (c)No restrictions on nationality or ethnicity (3). Intervention: studies using silver ion dressings, honey dressings, or ABC to treat DFUs (4). Control Group: studies with a control group receiving standard care (sterile gauze or povidone-iodine dressings) or other dressings for comparative purposes (5). Outcome Reporting: studies reporting healing rate, healing time or wound area reduction. Other outcomes: (a) Infection Status: only ulcers without active infection at baseline were included in studies. (b) Adverse events: including local skin irritation, allergic reactions, osteomyelitis, amputation, abnormal wound exudation, or systemic reactions related to the dressings. We excluded studies involving additional interventions (e.g., negative pressure therapy and hyperbaric oxygen therapy), as well as those in which treatment was administered via injection or other non-topical methods. The reference lists of relevant articles and reviews were also screened for additional studies. Studies involving participants with lower extremity venous ulcers, pressure ulcers or other non-DFUs ulcerative wounds were also excluded. The PRISMA 2020 flow diagram was downloaded from the official PRISMA website (https://www.prisma-statement.org/prisma-2020-flow-diagram) to illustrate the inclusion and exclusion process.

### 2.3 Data extraction

Two authors (Y.L. and J.L.) independently extracted data from the included studies, with disagreements resolved through consensus among all authors. The following data were collected from each eligible study: basic study characteristics (title, first author, publication year, Region, sample size, mean age, follow up, Wagner’s grade) and detailed PICO (Population, Intervention, Comparison, Outcome) information. Key outcome indicators—including healing rate, healing time, wound area reduction, infection status and adverse events—were required to be reported in the included literature. Wound closure was assessed by on-site investigators and defined as complete (100%) re-epithelialization without wound exudate. For duplicate studies or studies with overlapping data, the most comprehensive and recently published information was prioritized for inclusion.

### 2.4 Bias risk assessment

To assess the quality of the included studies, the Risk of Bias 2 (RoB2) tool was used for evaluating *in vivo* studies (13). The RoB2 tool consists of 10 entries that can be used to detect bias of selection, performance and so on. All studies were reported as low risk, unclear risk and high risk. When the conclusions of two reviewers differed significantly, they consulted with a senior researcher to make the final decision.

### 2.5 Statistical analysis

Network meta-analysis (NMA) was performed using Stata 18.0 software (https://www.stata.com) for the following steps: (1) A network evidence diagram was constructed to visualize the quantitative associations between different interventions (2). For networks containing closed loops, global and local inconsistency tests were conducted. If all comparison results yielded p > 0.05, the network was considered to have good consistency, and direct and indirect comparisons were deemed consistent. Loop inconsistency tests were further performed to evaluate the consistency of each closed loop for individual outcomes. If the 95% CI of the loop inconsistency factor (IF) included 0, direct and indirect evidence was considered consistent, and a consistency model was adopted; otherwise, an inconsistency model was used (3). Pairwise meta-analysis was performed by forest plots. The healing rate, a binary variable, we estimated the summary odds ratio (OR) with 95% confidence intervals (CIs). For continuous variables, including wound healing time and wound area reduction, were expressed as standardized mean difference (SMD) with 95%CIs (4). The surface under the cumulative ranking curve (SUCRA) indicates the relative ranking probability of each treatment, with higher SUCRA values representing better performance for positive outcomes and lower risk for negative outcomes (5). A comparison-adjusted funnel plot was created to detect publication bias and small-study effects in the intervention network. Publication bias was assessed using both funnel plot visualization and Egger’s test. Asymmetric distribution in the funnel plot indicated potential publication bias or small-study effects, and publication bias was confirmed if Egger’s test resulted in p < 0.05. Sensitivity analysis was performed via trim-and-fill analysis. The results were considered robust if the pooled effect size and corresponding p value showed no significant changes in magnitude or direction before and after the trim-and-fill procedure. The statistical significance level was set at α = 0.05.

## 3 Results

### 3.1 Study selection and characteristics

**Figure 1:**
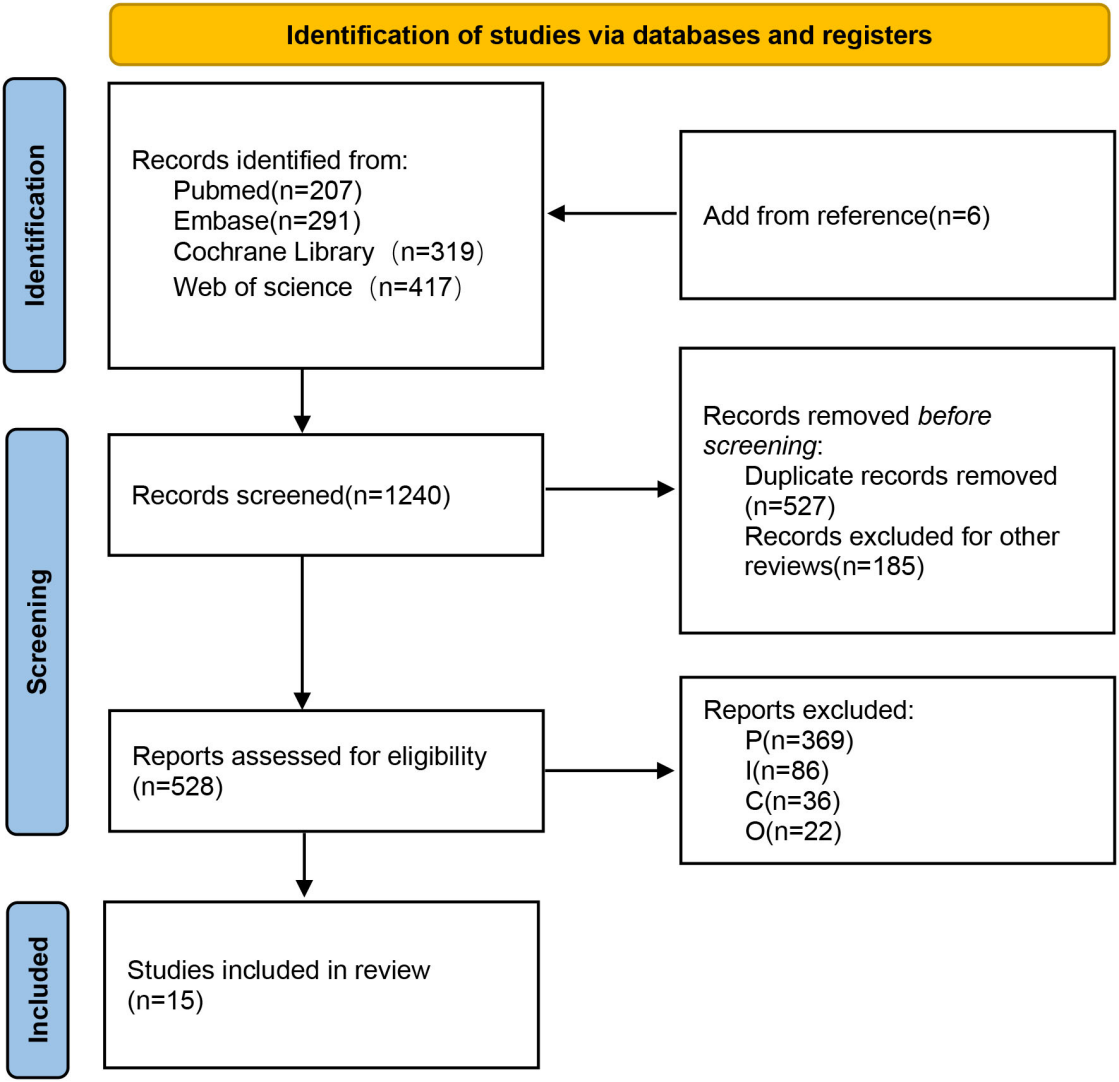
PRISMA flow diagram of the study processes.

We identified 1240 potentially eligible studies through the initial electronic searches. Ultimately, 15 studies (14–28) met the selection criteria for this review (**Figure 1**). The fundamental characteristics of studies and clinical trials are summarized in **Table 1**. The year of publication ranged from 2012 to 2024, with 12 originating from Asia, 1 from Africa, 1 from Europe and 1 from North America. A total of 1,292 patients with DFUs were included. The included studies encompassed 4 distinct interventions: SOC (Standard of Care), ABC (Antibacterial bone cement), Silver and Honey. Among them, there are 5 studies comparing Silver vs SOC, 5 studies on Honey vs SOC, 2 studies on ABC vs SOC, 2 studies on Silver vs Honey, 1 study on ABC vs Silver.

**Table 1:** Characteristics of studies included in the network meta-analysis.

Included trials	Region	Intervention	N	Age(y)(mean ± sd)	Comparison	N	Age(y)(mean ± sd)	Follow-up	Wagner’s grade	Outcomes
Cao, T.2023 (15)	China	ABC	12	64 ± 8	Silver	12	62 ± 8	3w	III,IV	①②
Essa, M. S.2023 (16)	Egypt	Silver	40	55.8 ± 2.38	SOC	40	54.64 ± 3.54	12w	\	①②③
He, W. Q.2016 (17)	China	Silver	40	55.7 ± 21.2	SOC	40	58.8 ± 21.9	\	\	①②③
Hosseinpoor, S.2023 (18)	Iran	Silver	24	58.92 ± 6.6	SOC	14	58.95 ± 5.6	4w	\	④
Imran, M.2015 (19)	Saudi Arabia	Honey	179	54 ± 4.25	SOC	169	54 ± 4	17w	\	①②
Kamaratos, A. V.2014 (20)	Greece	Honey	32	56 ± 14	SOC	31	57 ± 15	16w	\	①②④
Lafontaine, N.2023 (21)	Australia	Silver	63	61 ± 12	SOC	55	62 ± 14	12w	I,II	①②③④⑤
Searan, Wael Mahmoud.2024 (23)	Saudi Arabia	Honey	30	59.64 ± 10.21	SOC	30	59.64 ± 10.21	12w	\	②③
Tsang, Ka-Kit.2017 (27)	Hong Kong	Silver	11	63.36 ± 11.31	Honey	10	65.6 ± 11.42	12w	\	①③④⑤
Chao liu.2019 (22)	China	ABC	28	68.9 ± 9.1	SOC	22	67.8 ± 7.4	\	III,IV	①②⑤
Rajkumar Sharma.2017 (24)	India	Silver	12	58.23 ± 11.59	SOC	13	54.08 ± 10.33	12w	I,II	①③
Naeem Ullah.2012 (28)	Pakistan	Honey	50	56 + 8.0	SOC	50	56 + 8.0	10w	I-IV	①②⑤
Mansour Siavash.2013 (25)	Iran	Honey	32	60.00 ± 7	SOC	32	60.6 ± 7	12w	I-III	①②③
Mohammed Al Saeed.2019 (14)	Saudi Arabia	Honey	36	66.08 ± 12.9	Silver	35	66.43 ± 13.07	\	\	②
Sun Jie.2021 (26)	China	ABC	128	50.68 ± 8.23	SOC	22	51.37 ± 8.49	4w	\	①②④

Interventions: SOC, Standard of Care; ABC, Antibacterial bone cement. Outcomes indicators: ①healing rate; ②healing time; ③wound area reduction; ④Infection Status; ⑤adverse event.

### 3.2 Bias risk assessment

**Figure 2:**
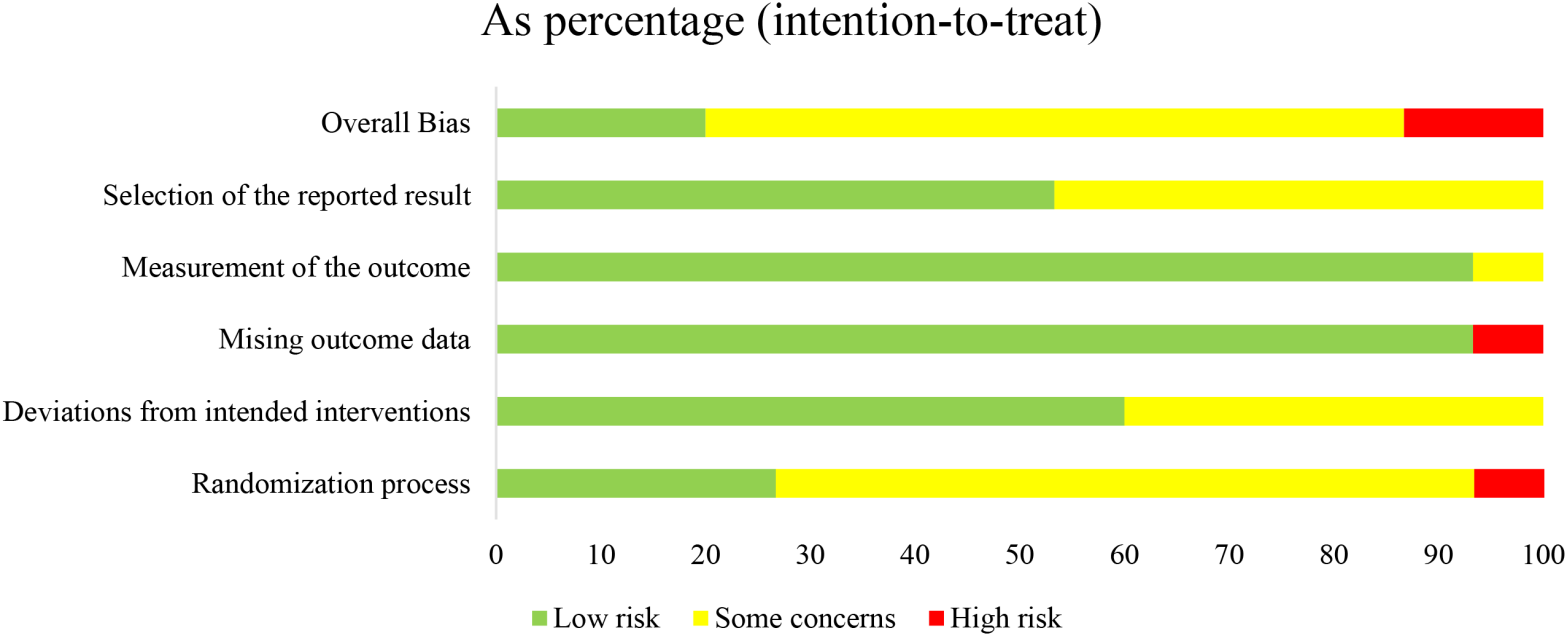
Bias risk assessment of the RCTs.

Assessment of risk of bias indicated that, among the 15 included studies, two studies (14, 26) did not specify the method of randomization, whereas the remaining 13 studies (15–25, 27, 28) provided a clear description of the randomization procedure: five studies used computer-generated random sequences, three employed block randomization, two applied alternating allocation, one used a random number table, one utilized convenience sampling, and one assigned dressings based on the type of treatment. Four studies (16, 17, 25, 27) explicitly reported the use of blinding during the study. Follow-up completeness was assessed based on the number of outcome events; 14 studies reported no loss to follow-up, indicating relatively complete data. Overall, the quality of the included studies was acceptable (**Figure 2**; **Supplementary Figure 1**).

### 3.3 Network evidence diagram

**Figure 3:**
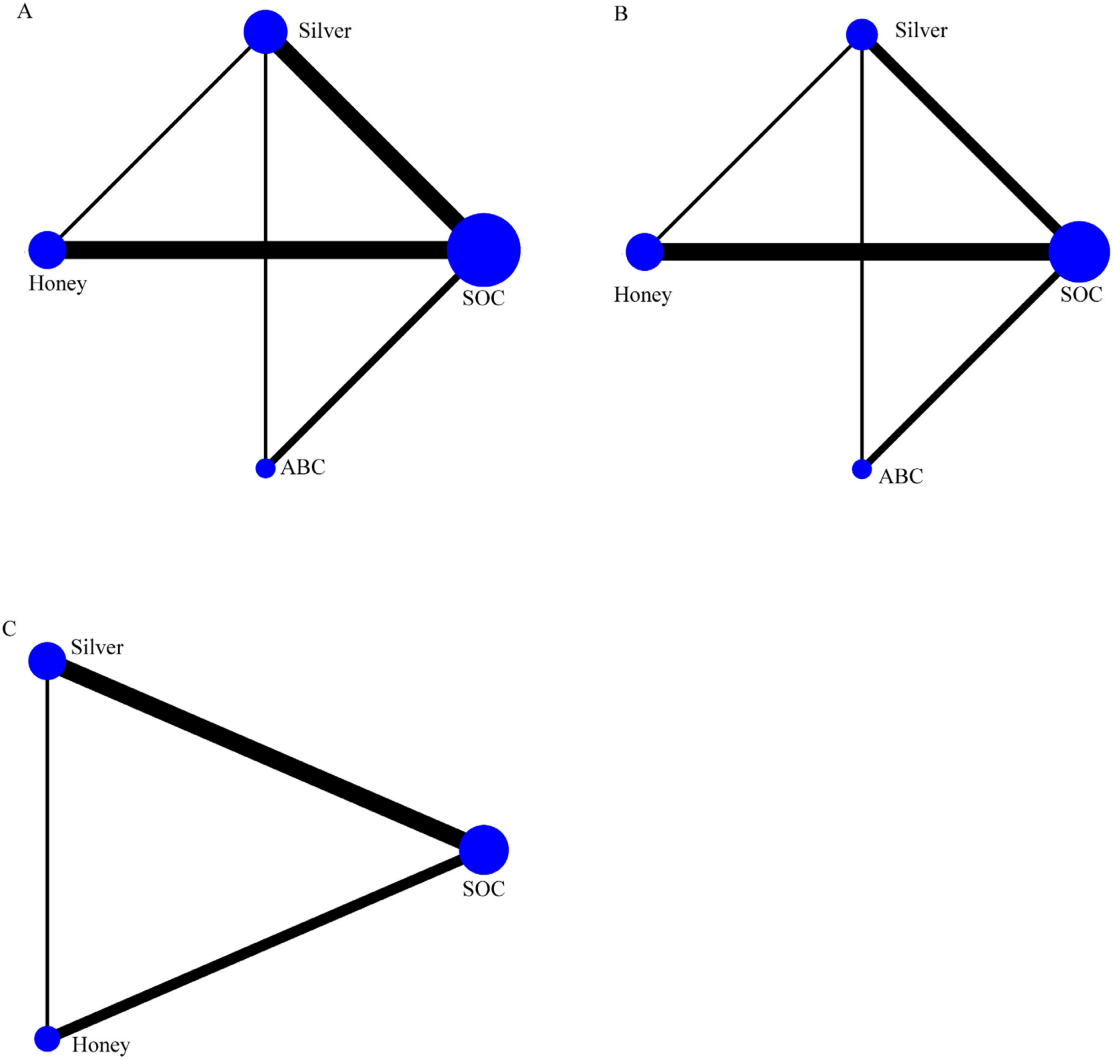
Network evidence plots for **(A)** healing rate, **(B)** healing time, **(C)** wound area reduction. The cirdes represent intervention arms in an RCT-larger cirdes represent presence in more RCTs. The lines connect interventions that were compared in an RCT and thicker connecting lines indicate more direct RCT comparisons.

Of the 15 included studies, 12 reported the healing rate of DFUs. Four interventions constituted a SOC-centered network diagram, forming two triangular closed loops (1): SOC − Honey − Silver (2); SOC – Silver −ABC (**Figure 3a**). Similarly, 12 studies documented the DFU healing time (**Figure 3b**). Additionally, 7 out of the 15 included studies reported DFU wound area reduction. Three interventions formed a SOC-centered network diagram, comprising one triangular closed loop: SOC − Honey – Silver (**Figure 3c**). In the network diagrams, node size denotes the number of interventions, while line thickness reflects the number of studies comparing the corresponding interventions.

### 3.4 Inconsistency test results

For the outcome of healing rate, the global inconsistency test yielded a p-value of 0.4881. For ulcer healing time and wound area reduction, the global inconsistency test returned p-values of 0.1854 and 0.5021, respectively. To further assess local inconsistency, the node-splitting method was employed, and the results showed that all pairwise comparisons had p-values > 0.05. Additionally, the 95% confidence intervals (CIs) of the inconsistency factors (IFs) included zero for all three outcomes.

### 3.5 NMA under the consistency

#### 3.5.1 Healing rate

**Figure 4:**
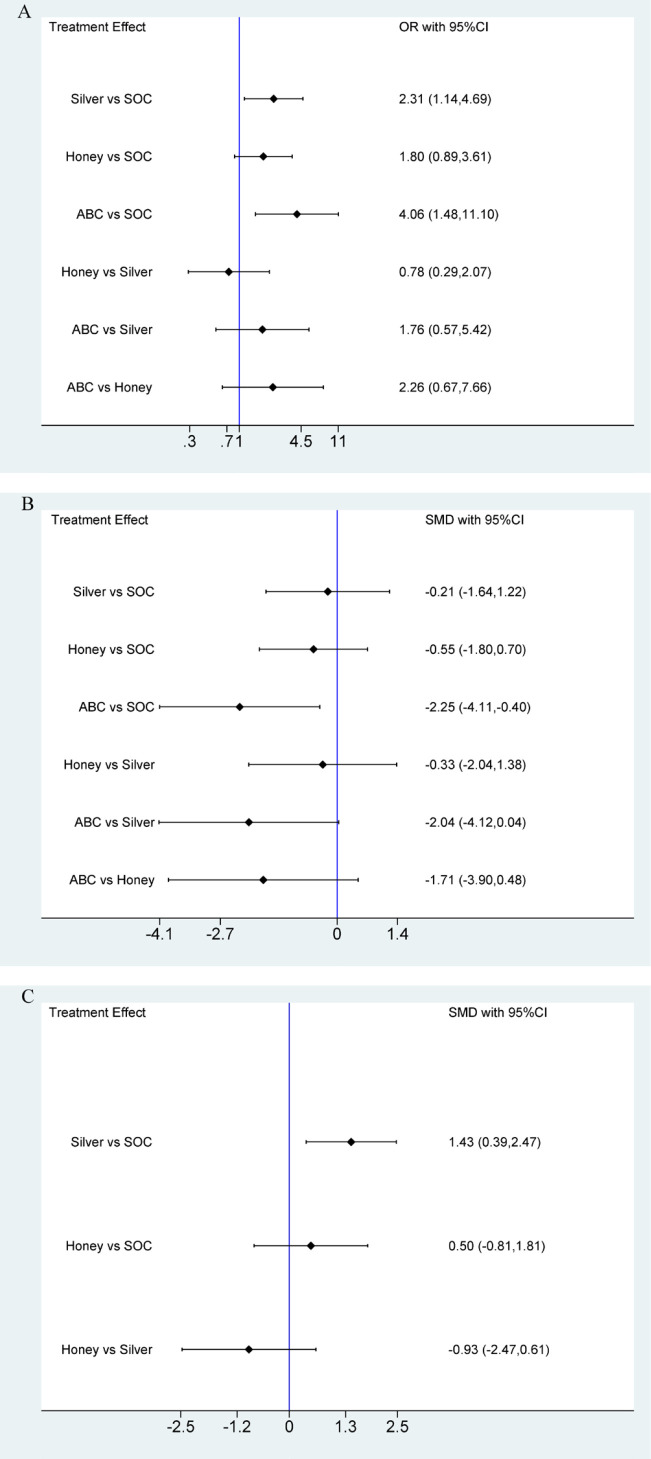
Forest plots for **(A)** healing rate; **(B)** healing time; **(C)** wound area reduction.

**Figure 5:**
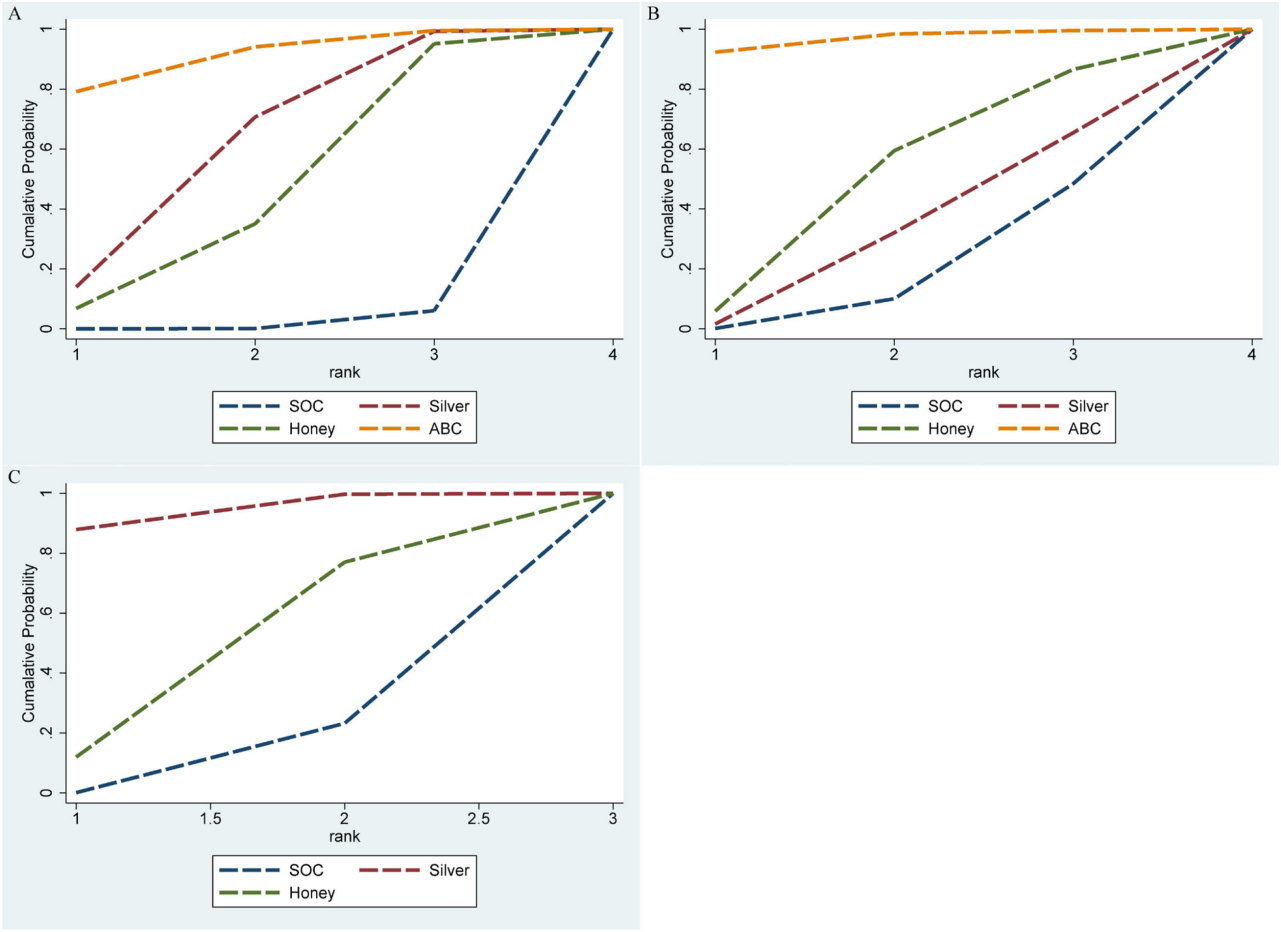
Cumulative ranking curves (SUCRA plots) for **(A)** healing rate, **(B)** healing time, **(C)** wound area reduction.

Twelve studies (15–17, 19–22, 24–28) involving 1,178 patients with DFU patients reported on the impact of the three antimicrobial dressings on complete wound healing rate. We observed that compared to SOC, ABC (OR = 4.06, 95% CI [1.48, 11.1], P < 0.05), silver (OR = 2.31, 95% CI [1.14, 4.69], P < 0.05) showed higher ulcer healing rates. No significant differences were observed among other interventions (**Figure 4a**; **Supplementary Figure 2**). The ranking of wound dressings based on SUCRA values was as follows: ABC (90.9%) > silver-containing dressings (61.3%) > honey dressings (45.7%) > standard care (2%) (**Figure 5a**).

#### 3.5.2 Healing time

Twelve studies (14–17, 19–23, 25, 26, 28) involving 1,253 patients with DFU patients reported on the impact of the three antimicrobial dressings on complete wound healing time. We observed that compared to SOC, ABC -2.25 (SMD =-2.25, 95% CI [-4.11, -0.40], P < 0.05) showed shorter ulcer healing time. No significant differences were observed among other interventions (**Figure 4b**; **Supplementary Figure 3**). The ranking of wound dressings based on SUCRA values was as follows: ABC (96.7%) > honey dressings (49.7%) > silver-containing dressings (33.4%) > standard care (20.2%) (**Figure 5b**).

#### 3.5.3 Wound area reduction

Seven studies (16, 17, 21, 23–25, 27) involving 503 patients with DFU patients reported on the impact of silver dressings and honey dressings on wound area reduction. We observed that compared to SOC, Silver -2.25 (SMD = 1.43, 95% CI [0.39, 2.47], P < 0.05) showed larger healing area. No significant differences were observed among other interventions (**Figure 4c**; **Supplementary Figure 4**). The ranking of wound dressings based on SUCRA values was as follows: silver-containing dressings (90.7%) > honey dressings (41.5%) > standard care (17.9%) (**Figure 5c**).

### 3.6 Infection status

Five studies (18, 20, 21, 26, 27) reported the anti-infective effects of silver-containing dressings, honey-based dressings, and antibiotic-loaded bone cement in the management of diabetic foot ulcers. Owing to heterogeneity in outcome measures and assessment criteria, only a descriptive analysis was performed. Among the two studies evaluating silver-containing dressings, the findings were inconsistent. Hosseinpoor et al. (18) demonstrated that, at 7, 14, and 21 days post-intervention, the infection rate in the silver dressing group was significantly lower than that in the standard care group. In contrast, Lafontaine et al. (21) observed no significant difference between the two groups at 12 weeks, which may be attributable to differences in follow-up duration and concomitant care strategies. Kamaratos et al. (20) confirmed that the proportion of sterile ulcers was higher in the honey dressing group than in the standard care group; however, evidence regarding the short- and long-term antimicrobial efficacy of honey-based dressings remains limited. Tsang et al. (27) simultaneously compared silver-containing dressings, honey-based dressings, and standard care, reporting that silver-containing dressings were superior in reducing wound microbial burden. In studies involving antibiotic-loaded bone cement, Sun et al. (26) identified the predominant pathogens associated with mild, moderate, and severe infections in diabetic foot ulcers, thereby providing a basis for antimicrobial agent and dressing selection. Nevertheless, the absence of post-intervention microbiological data precluded definitive conclusions regarding the infection control efficacy of antibiotic-loaded bone cement, underscoring the need for further investigation.

### 3.7 Adverse events

Four studies (21, 22, 27, 28) reported the occurrence of adverse events. The most commonly observed adverse events included osteomyelitis and amputation, as summarized in **Supplementary Table 4**.

### 3.8 Publication bias

A comparison-adjusted funnel plot was constructed using healing rates as an example (Supplementary **Figure 5**). The results indicate that the included studies were generally concentrated within the triangular region and symmetrically distributed around the central axis, suggesting that publication bias was essentially absent.

## 4 Discussion

DFU represents a severe complication of diabetes, with up to 28% of cases resulting in amputation or even death (29). The primary factors contributing to foot ulcers and tissue necrosis are ischemia, neuropathy, and infection (30). According to the International Working Group on the Diabetic Foot (IWGDF) and Infectious Diseases Society of America (IDSA) 2023 guidelines for the diagnosis and management of diabetic foot infections, Infection of a diabetic foot wound is defined as the presence of at least two of the classic signs or symptoms of inflammation (pain or tenderness, warmth, redness, swelling) or purulent secretions (pus) (31). Treatment of infection almost always requires antimicrobial therapy, which may be given systemically (to the whole body via the oral or parenteral (i.e. intravenous or intramuscular) route) or topically (i.e. locally, through application of antiseptic, antibiotic, or other antimicrobial preparations (e.g. solutions, creams, gels, ointments). A systematic review of antimicrobial agents for chronic wounds, including diabetic foot ulcers (DFUs), concluded that few systemic agents are associated with improved clinical outcomes, whereas the use of various topical agents may result in faster wound healing (32). Therefore, the appropriate selection and use of dressings in the management of diabetic foot ulcers may reduce the risk of infection, accelerate wound healing, and improve local tissue perfusion.

ABC demonstrates significant advantages in both healing rate and healing time, which closely aligns with its underlying therapeutic mechanism. The main component of bone cement is polymethyl methacrylate (PMMA), which offers excellent filling and scaffold properties, is highly biocompatible, and is widely employed in internal fixation, bone defect repair, and tissue cavity filling (33). In cases of severe DFU infection, systemic antibiotic therapy is typically required following debridement and necrotic tissue removal. However, systemic therapy often achieves insufficient local drug concentration, prolonging wound healing (34) and posing risks of adverse systemic effects. In contrast, ABC allows for prolonged local delivery of high-concentration antibiotics while minimizing systemic absorption, thereby significantly reducing the risk of systemic complications. Furthermore, ABC promotes autologous membrane formation, creating a relatively sealed environment that prevents bacterial invasion and provides a safe and stable microenvironment conducive to wound healing (33). Consequently, ABC not only shortens healing time but also improves the cure rate through growth factor release and biofilm inhibition. A systematic review encompassing 21 studies (35) demonstrated that ABC, compared to conventional therapies, significantly reduced wound healing time in DFU patients (SMD = -1.59, 95% CI [-2.04, -1.15], P < 0.001) and increased cure rates (OR = 5.26, 95% CI [3.03, 9.12], P < 0.001), consistent with the findings of the present study. The review also indicated that ABC reduced the time to bacterial clearance, although differences in amputation rates were not statistically significant. Evidence regarding the anti-infective efficacy of antibiotic-loaded bone cement was limited in our study. This may be attributable to the fact that standard care served as the comparator, whereas most previously published studies have used negative pressure wound therapy (NPWT) as the control intervention; accordingly, studies involving NPWT were excluded based on our predefined exclusion criteria. Two meta-analyses (36, 37) comparing antibiotic-loaded bone cement with NPWT demonstrated that the time to bacterial clearance was significantly shorter in the antibiotic-loaded bone cement group than in the NPWT group (MD = -7.30, 95% CI [-10.38, -4.32], p < 0.001; MD = −5.15, 95% CI [−7.15, −2.19], p < 0.001), confirming the superior antimicrobial efficacy of antibiotic-loaded bone cement. Currently, no consensus exists on optimal protocols for antibiotic selection or dosing in bone cement, especially when tailored to different anatomical sites or infection severities in DFU. This evidence gap highlights the need for further rigorous studies to establish a stronger foundation for the standardized and evidence-based use of antibiotic-loaded bone cement in clinical practice.

Silver ion dressings exhibited notable benefits in improving healing rate and reducing wound area, effects which can be attributed to their broad-spectrum antimicrobial and anti-biofilm properties, as well as their ability to maintain a conducive moist wound environment (38). Silver ions in dressings interact with bacterial DNA, enzymes, and proteins in the cell wall, disrupting cellular morphology, compromising membrane integrity, and ultimately inducing cell death (39). Additionally, silver ions promote fibroblast proliferation and differentiation into myofibroblasts, thereby accelerating wound closure (40). Two systematic reviews assessed the effectiveness of silver ion dressings in DFU (41, 42). One review (42), which included seven studies, concluded that silver ions improved healing rates (OR = 4.02, 95% CI [2.25, 7.17], P < 0.01) and reduced healing time (SMD = -2.81, 95% CI [-3.33, -2.29], P < 0.01). Due to the predominance of Chinese studies among the included literature, potential bias may exist, making the findings more applicable to Asian (especially Chinese) healthcare contexts and patient populations (42). Our analysis supports this view, indicating that the efficacy of silver dressings may not be fully generalizable to non-Asian populations. Future high-quality, multicenter randomized controlled trials conducted across diverse regions and healthcare settings are needed to comprehensively assess the global applicability of silver dressings.

Honey dressings, while not demonstrating clear superiority in healing rate, healing time, or wound area, significantly alleviate patient pain (43). Honey exhibits intrinsic antimicrobial activity and possesses osmotic properties that create a nutrient-rich, acidic-low, high-glucose microenvironment, stimulating macrophage activity and promoting wound repair while reducing discomfort (44). A systematic review of 16 studies (43) reported that honey dressings effectively shortened DFU healing time, increased healing rates (OR = 2.16, 95% CI [1.622–2.875], P < 0.00001), alleviated pain, reduced hospitalization duration, and enhanced granulation tissue formation. Nonetheless, current evidence remains insufficiently robust to draw definitive conclusions regarding honey’s efficacy in DFU management. Although no single antimicrobial dressing has been conclusively demonstrated to be superior, and benefits vary among dressings, clinical selection should consider ulcer infection status, wound size, local perfusion, patient comfort, and cost.

Our study conducted a network meta-analysis of 15 randomized controlled trials, encompassing 1,292 patients and four treatment modalities, to evaluate three novel dressings—silver ion, honey, and ABC—in terms of healing rate, cure rate, healing time, wound area, antimicrobial efficacy, and adverse events. Compared with standard care, ABC and silver ion dressings yielded higher cure rates; ABC shortened healing time; silver ion dressings achieved larger wound areas. Despite inconsistent evaluation standards for antimicrobial efficacy, silver ion dressings outperformed honey dressings in infection rate, bacterial clearance time, and bacterial reduction. Across the four treatments, the most common adverse events were osteomyelitis and amputation, with silver ion dressings effectively reducing these occurrences. Honey and ABC groups exhibited lower amputation rates among DFU patients.

The core innovation of this study lies in using network meta-analysis as a methodological tool to systematically integrate direct and indirect evidence of three antimicrobial dressings (including the cross-scenario application of ABC) for the first time. It fills the evidence gap regarding the relative efficacy and safety of dressings for DFU and achieves the transformation from “evidence integration” to “precision clinical application” through stratified recommendations and mechanism-linkage analysis. The 15 included studies indicate that ABC and silver ion dressings confer certain advantages; however, dressing selection should still be tailored to individual patient conditions.

This study has several limitations. First, 12 out of the 15 included studies (80%) were conducted in Asia. We acknowledge that regional differences exist in the incidence of diabetic foot ulcers, individual economic conditions, and healthcare resources. In Europe and North America, early interventions such as vascular reconstruction and systemic treatment are often prioritized, which may explain the observed geographical concentration in the application of antimicrobial dressings. Methodologically, most studies lacked blinding, which is a common challenge in wound care trials where masking is often unfeasible. Furthermore, the limited data volume from the included studies precluded subgroup analyses based on dressing subtypes or ulcer severity grades. Finally, a lack of economic data hindered a cost-effectiveness evaluation, which is essential for clinical decision-making.

## 5 Conclusions

In summary, the network meta-analysis of three antimicrobial dressings in DFU treatment demonstrates that ABC and silver ion dressings represent superior therapeutic options. Given the generally low quality of the included studies, future large-scale, multicenter trials are needed to enable direct comparisons and subgroup analyses of different antimicrobial dressings, with an emphasis on incorporating cost-effectiveness assessments to inform clinical decision-making.

## Data Availability

The original contributions presented in the study are included in the article/**Supplementary Material**. Further inquiries can be directed to the corresponding authors.

## References

[1] Ceriello A Colagiuri S . IDF global clinical practice recommendations for managing type 2 diabetes - 2025. Diabetes Res Clin practice. (2025) 222 Suppl 1:112152. doi: 10.1016/j.diabres.2025.112152, PMID: 40204549

[2] Chinese Society of Edndocrinology CEaMSA . Expert consensus on wound treatment of diabetic foot ulcer(2024). Chin J Endocrinol Metab. (2024) 40(7):565–9. doi: 10.3760/cma.j.cn311282-20240625-00281

[3] Lokeesan V Finlayson KJ Edwards HE Parker CN . Implementation of evidence-based practice in diabetic foot ulcer care in healthcare settings: A systematic review. Int J Nurs practice. (2025) 31:e70080. doi: 10.1111/ijn.70080, PMID: 41177159

[4] Chinese Diabeles Sociely CSoID Chinese Sociely for Tissue Repair . Chinese guideline on prevention and management of diabetic foot (2019). Chin J Diabetes Mellitus. (2019) 11:92–108. doi: 10.3760/cma.j.issn.1674-5809.2019.04.004

[5] Chen P Vilorio NC Dhatariya K Jeffcoate W Lobmann R McIntosh C . Guidelines on interventions to enhance healing of foot ulcers in people with diabetes (IWGDF 2023 update). Diabetes/metabolism Res Rev. (2024) 40:e3644. doi: 10.1002/dmrr.3644, PMID: 37232034

[6] Liu YF Ni PW Huang Y Xie T . Therapeutic strategies for chronic wound infection. Chin J traumatology = Zhonghua chuang shang za zhi. (2022) 25:11–6. doi: 10.1016/j.cjtee.2021.07.004, PMID: 34315658 PMC8787234

[7] Bajuri MY Nordin A . Activated carbon cloth versus silver-based dressings in a population with diabetic foot ulcer: a randomised controlled trial. J Wound Care. (2024) 33:298–303. doi: 10.12968/jowc.2024.33.5.298, PMID: 38683771

[8] Tajdar Y Singh S Raj A Raj A Bhushan V . Effect of silver colloid dressing over conventional dressings in diabetic foot ulcer: A prospective study. Turkish J surgery. (2024) 40:28–35. doi: 10.47717/turkjsurg.2024.6168, PMID: 39036009 PMC11257724

[9] Rosli MA Mohd Nasir NA Mustafa MZ Othman MA Zakaria Z Halim AS . Effectiveness of stingless bee (Kelulut) honey versus conventional gel dressing in diabetic wound bed preparation: A randomized controlled trial. J Taibah Univ Med Sci. (2024) 19:209–19. doi: 10.1016/j.jtumed.2023.11.003, PMID: 38124990 PMC10730855

[10] Lachiewicz PF Wellman SS Peterson JR . Antibiotic cement spacers for infected total knee arthroplasties. J Am Acad Orthopaedic Surgeons. (2020) 28:180–8. doi: 10.5435/JAAOS-D-19-00332, PMID: 31688429

[11] Ferrao P Myerson MS Schuberth JM McCourt MJ . Cement spacer as definitive management for postoperative ankle infection. Foot ankle Int. (2012) 33:173–8. doi: 10.3113/FAI.2012.0173, PMID: 22734277

[12] Dai J Zhou Y Mei S Chen H . Application of antibiotic bone cement in the treatment of infected diabetic foot ulcers in type 2 diabetes. BMC musculoskeletal Disord. (2023) 24:135. doi: 10.1186/s12891-023-06244-w, PMID: 36810078 PMC9942328

[13] Yang ZR Sun F Zhan SY . Risk of bias assessment: (1) overview. Zhonghua liu xing bing xue za zhi = Zhonghua liuxingbingxue zazhi. (2017) 38:983–7. doi: 10.3760/cma.j.issn.0254-6450.2017.07.027, PMID: 28738479

[14] Al Saeed M . Prospective randomized comparison of controlled release ionic silver hydrophilic dressings and medicated honey-impregnated dressings in treating neuropathic diabetic foot ulcer. Saudi J Health Sci. (2019) 8:25–30. doi: 10.4103/sjhs.sjhs_111_18

[15] Cao T Ji P Zhang Z Xiao D Wang KJ Li N . A prospective randomized controlled study of antibiotic bone cement in the treatment of diabetic foot ulcer. Zhonghua shao shang yu chuang mian xiu fu za zhi. (2023) 39:3110. doi: 10.3760/cma.j.cn501225-20221111-00485, PMID: 37805732 PMC11630292

[16] Essa MS Ahmad KS Zayed ME Ibrahim SG . Comparative study between silver nanoparticles dressing (SilvrSTAT gel) and conventional dressing in diabetic foot ulcer healing: a prospective randomized study. Int J lower extremity wounds. (2023) 22:4810. doi: 10.1177/1534734620988217, PMID: 33686887

[17] He WQ Luo WH Li L Jiang L . Effects of silver ions dressing for diabetic foot ulcers: a randomized controlled trial. Chin J Evidence-Based Med. (2016) 16:5100. doi: 10.7507/1672-2531.20160079

[18] Hosseinpoor S Kalroozi F Nezamzadeh M Pishgooie SA . Examining the effect of polyurethane dressing containing silver particles on the rate of diabetic foot ulcer infection in hospitalized patients: A randomized control study. Health Sci Rep. (2023) 6(11):e1733. doi: 10.1002/hsr2.1733, PMID: 38028699 PMC10680056

[19] Imran M Hussain MB Baig MA . A Randomized, Controlled Clinical Trial of Honey-Impregnated Dressing for Treating Diabetic Foot Ulcer. J Coll Physicians Surgeons–Pakistan. (2015) 25:721. doi: 10.2015/JCPSP.721725, PMID: 26454386

[20] Kamaratos AV Tzirogiannis KN Iraklianou SA Panoutsopoulos GI Kanellos IE Melidonis AI . Manuka honey-impregnated dressings in the treatment of neuropathic diabetic foot ulcers. Int Wound J. (2014) 11:259. doi: 10.1111/j.1742-481X.2012.01082.x, PMID: 22985336 PMC7950714

[21] Lafontaine N Jolley J Kyi M King S Iacobaccio L Staunton E . Prospective randomised placebo-controlled trial assessing the efficacy of silver dressings to enhance healing of acute diabetes-related foot ulcers. Diabetologia. (2023) 66:768eded. doi: 10.1007/s00125-022-05855-7, PMID: 36629877

[22] Liu C You JX Chen YX Zhu WF Wang Y Lv PP . Effect of induced membrane formation followed by polymethylmethacrylate implantation on diabetic foot ulcer healing when revascularization is not feasible. J Diabetes Res. (2019) 2019:2429136. doi: 10.1155/2019/2429136, PMID: 31828156 PMC6885796

[23] Searan WM Abdalqader MA Al-Goshae HA Nor HM Al-Shubrumi HS Badahdah H . The influence of honey and hydrogel products therapy on healing time in diabetic foot. Int J Lower Extremity Wounds. (2024) 29:15347346241233236. doi: 10.1177/15347346241233236, PMID: 38425229

[24] Sharma R Gupta N Kumar V Pal S Kaundal V Sharma V . Silver colloid dressings score over conventional dressings in diabetic foot ulcer: a randomized clinical trial. Int Surg J. (2017) 4:2627–31. doi: 10.18203/2349-2902.isj20173401

[25] Siavash M Shokri S Haghighi S Shahtalebi MA Farajzadehgan Z . The efficacy of topical royal jelly on healing of diabetic foot ulcers: a double-blind placebo-controlled clinical trial. Int Wound J. (2013) 12:137–42. doi: 10.1111/iwj.12063, PMID: 23566071 PMC7950674

[26] Jie Zl S Ll Hongye Y Jia-Xing DG-C . Etiological characteristics of diabetic foot infection and therapeutic effect ofvancomycin combined with bone cement iniection. Chin J Nosocomiol. (2021) 31:24–7. doi: 10.11816/cn.ni.2021-201709

[27] Tsang K-K Kwong EW-Y To TS-S Chung JW-Y Wong STK . A pilot randomized, controlled study of nanocrystalline silver, manuka honey, and conventional dressing in healing diabetic foot ulcer. Evidence-Based Complementary Altern Med. (2017) 2017:5294890. doi: 10.1155/2017/5294890, PMID: 28239398 PMC5296609

[28] Habibullah UN Khan K Khan A Ashfaq-ur-rahman BK . Comparison of conventional pyodine dressing with honey dressing for the treatment of diabetic foot ulcers. J Pak Orthop Assoc. (2012) 23:89–94.

[29] Bus SA Van Netten JJ Hinchliffe RJ Apelqvist J Lipsky BA Schaper NC . Standards for the development and methodology of the 2019 International Working Group on the Diabetic Foot guidelines. Diabetes/metabolism Res Rev. (2020) 36 Suppl 1:e3267. doi: 10.1002/dmrr.3267, PMID: 31916377

[30] Branch Group of Diabetic Foot and Peripheral Vascular Disease CDS . Clinical pathway for the management of diabetic foot in China(2023). Chin J Endocrinol. (2023) 39:93–102. doi: 10.3760/cma.j.cn311282-20221014-00583

[31] Senneville É Albalawi Z van Asten SA Abbas ZG Allison G Aragón-Sánchez J . IWGDF/IDSA guidelines on the diagnosis and treatment of diabetes-related foot infections (IWGDF/IDSA 2023). Diabetes/metabolism Res Rev. (2024) 40:e3687. doi: 10.1002/dmrr.3687, PMID: 37779323

[32] Dumville JC Lipsky BA Hoey C Cruciani M Fiscon M Xia J . Topical antimicrobial agents for treating foot ulcers in people with diabetes. Cochrane Database systematic Rev. (2017) 6:Cd011038. doi: 10.1002/14651858.CD011038.pub2, PMID: 28613416 PMC6481886

[33] Chen H Yao L Zhou Y Dai J . Evaluation of antibiotic-loaded bone cement in treatment of infected diabetic foot: systematic review and meta-analysis. Diabetes/metabolism Res Rev. (2024) 40:e70002. doi: 10.1002/dmrr.70002, PMID: 39497440

[34] Gouron R Petit L Boudot C Six I Brazier M Kamel S . Osteoclasts and their precursors are present in the induced-membrane during bone reconstruction using the Masquelet technique. J Tissue Eng regenerative Med. (2014) 11:382–9. doi: 10.1002/term.1921, PMID: 24919776

[35] Zhao Yamei FL Bin X Yan C Jian W . Meta-analysis of the efficacy of antibiotic bone cement in treating diabetic foot ulcers. Chin J Diabetes Mellitus. (2024) 16:790–8. doi: 10.1016/j.heliyon.2023.e23398, PMID: 38226271 PMC10788428

[36] Dong T Huang Q Sun Z . Antibiotic-laden bone cement for diabetic foot infected wounds: A systematic review and meta-analysis. Front endocrinology. (2023) 14:1134318. doi: 10.3389/fendo.2023.1134318, PMID: 37008902 PMC10060955

[37] Wu S Xu Y Guo L Jiang X . A meta-analysis of the effectiveness of antibacterial bone cement in the treatment of diabetic foot skin wound infections. Int Wound J. (2024) 21:e14487. doi: 10.1111/iwj.14487, PMID: 37973553 PMC10898415

[38] Chauhan A Patel B Sharma PP FsJIJoCS M . A prospective, randomized, controlled study for efficacy of phenytoin sodium powder, eusol solution, nanocrystalline silver gel in diabetic foot ulcer. (2019) doi: 10.5958/2321-1024.2019.00014.X

[39] Lin H BoLatai A Wu N . Application progress of nano silver dressing in the treatment of diabetic foot. Diabetes Metab syndrome obesity: Targets Ther. (2021) 14:4145–54. doi: 10.2147/DMSO.S330322, PMID: 34621128 PMC8491782

[40] Liu X Lee PY Ho CM Lui VC Chen Y Che CM . Silver nanoparticles mediate differential responses in keratinocytes and fibroblasts during skin wound healing. ChemMedChem. (2010) 5:468–75. doi: 10.1002/cmdc.200900502, PMID: 20112331

[41] Hosseini RS Hasanpour K Khoshnevis M Fakhr MS Derin E Ghaffarian A . Therapeutic effect of silver nanoparticles in the management of diabetic ulcers: A systematic review and meta-analysis on RCTs. Int J lower extremity wounds. (2024) 15347346241241836. doi: 10.1177/15347346241241836, PMID: 38544426

[42] Luo Y Li L Zhao P Yang C Zhang J . Effectiveness of silver dressings in the treatment of diabetic foot ulcers: a systematic review and meta-analysis. J Wound Care. (2022) 31:979–86. doi: 10.12968/jowc.2022.31.11.979, PMID: 36367799

[43] Yildiz Karadeniz E Kaplan Serin E . Use of honey in diabetic foot ulcer: Systematic review and meta-analysis. J Tissue Viability. (2023) 32:270–8. doi: 10.1016/j.jtv.2023.03.002, PMID: 37032304

[44] Kateel R Adhikari P Augustine AJ Ullal S . Topical honey for the treatment of diabetic foot ulcer: A systematic review. Complementary therapies Clin practice. (2016) 24:130–3. doi: 10.1016/j.ctcp.2016.06.003, PMID: 27502813

